# Prenatal Exposure to Lipopolysaccharide Combined with Pre- and Postnatal High-Fat Diet Result in Lowered Blood Pressure and Insulin Resistance in Offspring Rats

**DOI:** 10.1371/journal.pone.0088127

**Published:** 2014-02-03

**Authors:** Xue-Qin Hao, Jing-Xia Du, Yan Li, Meng Li, Shou-Yan Zhang

**Affiliations:** 1 Department of Pharmacy, College of Animal Science and Technology, Henan University of Science and Technology, Luoyang, PR China; 2 Department of pharmacology, Medical College, Henan University of Science and Technology, Luoyang, PR China; 3 Luoyang Entry-Exit Inspection and Quarantine Bureau, Luoyang, PR China; 4 Department of Cardiology, Luoyang Central Hospital Affiliated to Zhengzhou University, Luoyang, PR China; Universidad Pablo de Olavide, Centro Andaluz de Biología del Desarrollo-CSIC, Spain

## Abstract

**Background:**

Adult metabolic syndrome may in part have origins in fetal or early life. This study was designed to explore the effect of prenatal exposure to lipopolysaccharide and high-fat diet on metabolic syndrome in offspring rats.

**Methods:**

32 pregnant rats were randomly divided into four groups, including Control group; LPS group (pregnant rats were injected with LPS 0.4 mg/kg intraperitoneally on the 8^th^, 10^th^ and 12^th^ day of pregnancy); High-fat group (maternal rats had high-fat diet during pregnancy and lactation period, and their pups also had high-fat diet up to the third month of life); LPS + High-fat group (rats were exposed to the identical experimental scheme with LPS group and High-fat group).

**Results:**

Blood pressure elevated in LPS group and High-fat group, reduced in LPS+High-fat group, accompanied by the increase of serum leptin level in LPS and High-fat group and increase of serum IL-6, TNF-a in High-fat group; both serum insulin and cholesterol increased in High-fat and LPS+High-fat group, as well as insulin in LPS group. HOMA-IR value increased in LPS, High-fat and LPS+High-fat group, and QUICKI decreased in these groups; H-E staining showed morphologically pathological changes in thoracic aorta and liver tissue in the three groups. Increased serum alanine and aspartate aminotransferase suggest impaired liver function in LPS+High-fat group.

**Conclusion/Significance:**

Prenatal exposure to lipopolysaccharide combined with pre- and postnatal high-fat diet result in lowered blood pressure, insulin resistance and impaired liver function in three-month old offspring rats. The lowered blood pressure might benefit from the predictive adaptive response to prenatal inflammation.

## Introduction

The pathophysiology of Type 2 diabetes (T2DM) is characterized by a low-grade chronic inflammation, with the release of inflammatory cytokines by innate immune cells (mainly macrophages and dendritic cells) that impair insulin action [1]. Accumulating evidence from animal studies suggest that chronic elevation of circulating lipopolysaccharide (LPS), a key component of gram negative bacteria cell walls, is considered to be a causative factor for insulin resistance [2]. Obese and T2DM subjects have elevated LPS concentrations in the circulation, and LPS directly inhibits insulin signaling and glucose transport in human muscle cells. Pharmacological and genetic inhibition of LPS-induced inflammation leads to enhanced insulin action [2]. It suggests that inflammation is a mechanism connected with the risk of type 2 diabetes [3].

A fat-enriched diet favors the development of gram negative bacteria in the intestine which is linked to the occurrence of T2DM. It was suggested that the intestinal microbiota contributes to the development of obesity and insulin-resistance. A switch from a normal diet towards a fat-enriched diet, where the daily amount of dietary fibers is reduced, was associated with a change in the ecology of the intestinal microbiota with an increase in gram-negative bacteria [1]. An increase in plasma LPS occurs in healthy individuals after a high-fat meal, whereas a chronic state of low-grade endotoxemia as measured by plasma LPS is evident in patients with obesity and insulin resistance [4].

A great deal of evidence have demonstrated the link between fetal and postnatal growth and development of adult cardiovascular risk factors including hypertension, dyslipidemia, obesity, altered vascular endothelial function and glucose homeostasis. It suggests that adult metabolic syndrome may in part have origins in fetal or early life [5]. Population-based studies suggest that fetal adaptive responses to maternal dietary imbalance confer survival benefit when the postnatal diet remains suboptimal but increase susceptibility to cardiovascular disease when postnatal nutrition is improved [6]. Predictive adaptive responses are also observed in adverse environmental conditions, for example, sweat gland density is determined in early postnatal life by environmental temperature, an adaptation that may determine the degree of tolerance to extremes of climate later in life [6], [7]. In addition, fathers can also initiate intergenerational transmission of obesity/metabolic diseases, induced indirectly or directly, such as through exposure to a high-fat diet [8].

The link between maternal high-fat diet and metabolic diseases has been well recognized [9]-[11], and previous studies have also showed that prenatal exposure to LPS (0.79 mg/kg) programs hypertension, obesity and insulin resistance in offspring rats [12]–[14]. But the influence of prenatal LPS exposure combined with pre- and early postnatal high-fat diet on metabolic diseases in offspring rats was not known. May they create a predictive additive effect due to the prenatal inflammation caused by both LPS treatment and high-fat diet, or chronic high-fat diet relative to acute LPS injection intraperitoneally may produce a benefit effect on offspring as a predictive adaptive response? This study was designed to explore the effect of prenatal LPS (0.4 mg/kg) exposure combined with pre- and early postnatal high-fat diet on metabolic syndrome in three-month old offspring rats.

## Materials and Methods

### Animals

Sixty Sprague-Dawley rats (40 females and 20 males) were purchased from Animal Center of Tongji Medical College, Huazhong University of Science and Technology (Wuhan, China). All animals had free access to standard laboratory rat chow and tap water in a room at constant temperature (24°C) and under a 12 h light-dark cycle. After acclimation for two weeks, the males were with the females for 15 hours, and day 0 of pregnancy was confirmed the next morning by the presence of a vaginal plug. After parturition, pups were raised with a lactating mother until 4 weeks of age, at which time they were removed to cages containing four rat pups. The present study was conducted in accordance with the principles outlined in the National Institutes of Health (NIH) *Guide for the Care and Use of Laboratory Animals* (http://grants1.nih.gov/grants/olaw/) and was approved by the local animal ethics committee at Henan University of Science and Technology.

### Dams and litters

The pregnant rats were randomly divided into four groups (n = 8 in each): Control group, LPS group, High-fat group and LPS+High-fat group. The rats in Control group and LPS group had normal diet, and were intraperitoneally administered with vehicle, 0.40 mg/kg LPS (Sigma Chemical, St Louis, MO, USA) respectively on the 8^th^, 10^th^ and 12^th^ day of pregnancy. Rats in High-fat group were exposed to high-fat diet (based on the normal diet, added 10% lard oil, 5% cholesterin, 1.5% bile salt from pig, 10 egg yolks/kg, 0.14 kg milk powder/kg, some sugar and trace elements) during pregnancy and lactation period, and their pups also had High-fat diet up to the third month of life. Rats in LPS+High-fat group were exposed to the identical experimental scheme with LPS group and High-fat group.

### Blood collection

Rats fasted for 12 h, were anesthetized with pentobarbital (40 mg/kg). Blood was taken from abdominal aorta and put at room temperature for 30 minutes, then was centrifuged at 3000 r/min for 10 minutes. The supernatant serum was taken and stored at −20°C.

### Measurement of serum IL-6, TNF-a and leptin concentration

Serum IL-6, TNF-a and leptin concentrations were measured with Enzyme-linked immunosorbent assay (Elisa) method using Rat IL-6, TNF-a and leptin ELISA kits (Shanghai Resun Biological Technology Co., Ltd., Shanghai, China) according to the instructions. The microplates were read using a SpectraMax M5 microplate reader (SpectraMax M5, US).

### Systolic blood pressure measurement

Blood pressure was measured with Carotid Artery Intubation method using BL-420E Biological Signal Acquisition System (Chengdu Thai Union Electronics Co., Ltd., Chengdu, China) when offspring rats were three-month old. The offspring rats were anesthetized with pentobarbital (40 mg/kg) and put on the table; then carotid artery was exposed, intubated and connected to BL-420E Biological Signal Acquisition System. The average systolic blood pressure was recorded.

### Measurement of serum glucose and insulin

Serum glucose was measured with Glucose Oxidase method using Glucose assay kit (Sichuan new biological technology Co., Ltd., Chengdu, China) and TBA-2000FR automatic biochemical analyzer (Toshiba Medical Systems Co., Ltd., Japan). Serum insulin was measured with Chemiluminescence method using Insulin determination kit (Tianjin Bo oasis Biological Technology Co., Ltd., Tianjin, China) and TBA-2000FR automatic biochemical analyzer (Toshiba Medical Systems Co., Ltd., Japan).

### Measurement of serum triglyceride and cholesterol

Serum triglyceride and cholesterol were measured with Oxidase method using Triglyceride assay kit and Cholesterol assay kit (Intec Biotechnology Co., Ltd., Xiamen, China), respectively, and detected with TBA-2000FR automatic biochemical analyzer (Toshiba Medical Systems Co., Ltd., Japan).

### Calculation of HOMA-IR and QUICKI

HOMA-IR was calculated according to the formulas below:
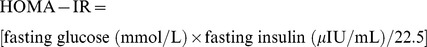



Insulin sensitivity was estimated using the Quantitative Insulin Sensitivity Check Index (QUICKI) according to equation: QUICKI =  1/[(log insulin (μIU/mL) + log glucose (mg/dL)]. Low QUICKI indicates low insulin sensitivity, while high QUICKI indicates high insulin sensitivity.

### Evaluation of liver function

Liver function was evaluated by serum levels of alanine aminotransferase, aspartate aminotransferase, total bilirubin, total protein and albumin. Alanine aminotransferase and aspartate aminotransferase were determined with IFCC method using Alanine aminotransferase and Aspartate aminotransferase determination kits (Sichuan New Biological Technology Co., Ltd., Chengdu, China), respectively. Total bilirubin was measured with Vanadate Oxidation method using Total bilirubin assay kit (Sichuan New Biological Technology Co., Ltd., Chengdu, China). Total protein was determined with Biuret method using Total protein assay kit (Intec Biotechnology Co., Ltd., Xiamen, China); albumin was determined with Bromocresol Green Colorimetry method using Albumin assay kit (Intec Biotechnology Co., Ltd., Xiamen, China). All these indexes were detected with TBA-2000FR automatic biochemical analyzer (Toshiba Medical Systems Co., Ltd., Japan).

### Evaluation of renal function

Renal function was evaluated by serum levels of urea nitrogen, creatinine and uric acid. Serum urea nitrogen was determined with UV-Glutamate Dehydrogenase method using Kit for determination of urea nitrogen; serum creatinine was determined with Sarcosine Oxidase method using Creatinine assay kit; serum uric acid was determined with Oxidase method using Uric acid assay kit. All these kits were purchased from Sichuan New Biological Technology Co., Ltd., Chengdu, China. These indexes were detected with TBA-2000FR automatic biochemical analyzer (Toshiba Medical Systems Co., Ltd., Japan).

### Morphological changes of thoracic aorta and liver tissue

After perfusion with 0.9% Nacl and 4% paraformaldehyde, thoracic aorta and liver tissues were collected and incubated in 4% paraformaldehyde solution for 48 h, then dehydrated and embedded in paraffin wax; tissue was sliced into sections (4 µm) and HE staining was performed. Morphological changes of thoracic aorta and liver tissue were observed under light microscope.

### Statistical analysis

Results are presented as means ± SEM. One-way ANOVA followed by Tukey's post hoc test was used to assess the statistical significance between groups. Two-way ANOVA followed by Bonferroni's post-test was used to assess the interaction between prenatal LPS exposure and high-fat diet on the values. P<0.05 was considered significant. All analyses were performed with SPSS 13.0 (SPSS Inc., Chicago, IL, USA).

## Results

### Delivery rate

Delivery rates for different groups are 10±2 puppies per litter in Control group and LPS group; 8±2 puppies per litter in High-fat group and LPS + High-fat group.

### Food intake

Food intake of pregnant rats in Control group and LPS group was 46.3±4.2 g and 45.6±4.5 g per day; Food intake of pregnant rats in High-fat group and LPS+High-fat group was 29.3±3.5 g, 27.7±3.3 g per day, respectively.

Food intake of 3 month-old male offspring rats in Control group and LPS group was 34.7±3.4 g, 35.3±2.8 g per day, in female offspring rats was 26.6±1.3 g and 27.0±1.8 g, respectively. Food intake of 3 month-old male offspring rats in High-fat and LPS+High-fat group was 20.4±2.7 g, 19.0±2.5 g per day, in female offspring rats was 16.3±1.3 g and 15.5±1.5 g, respectively.

### Serum IL-6, TNF-a and leptin concentration

**Figure 1:**
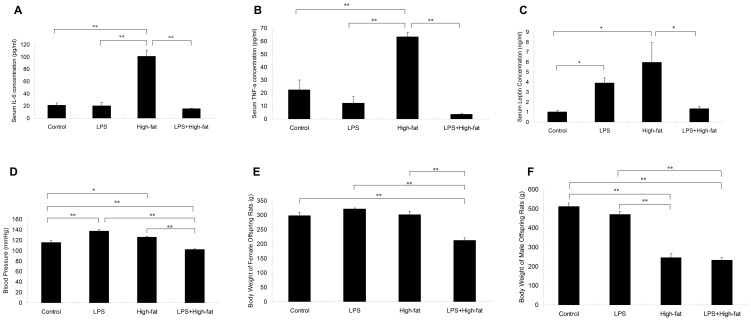
Effects of prenatal exposure to lipopolysaccharide combined with pre- and postnatal high-fat diet on serum IL-6 (A), TNF-a (B), leptin (C), blood pressure (D) and body weight (E, F) in three-month old offspring rats. Data are presented as the mean ± SEM (n = 8 in each group). ^**^ *P*<0.01, ^*^ *P*<0.05 (one-way ANOVA).

Serum IL-6 and TNF-a concentration in High-fat group increased significantly compared with Control group, LPS group and LPS+High-fat group (*p*<0.01) (Figure 1A and 1B). There was no significant difference between Control group, LPS group and LPS+High-fat group. Serum leptin concentration in High-fat group increased significantly compared with Control group, LPS group and LPS+High-fat group (*p*<0.05) (Figure 1C). Significant interaction was found between prenatal LPS exposure and pre- and postnatal high-fat diet on serum IL-6 (F = 38.822, df = 1, *p*<0.01), TNF-a (F = 13.801, df = 1, *p*<0.01) and leptin concentration (F = 36.017, df = 1, *p*<0.01).

### Systolic blood pressure

Systolic blood pressure in LPS group (*p*<0.01) and High-fat group (*p*<0.05) increased significantly compared with Control group, while systolic blood pressure in LPS+High-fat group decreased significantly compared with Control, LPS and High-fat groups (*p*<0.01). Significant interaction was found between prenatal LPS exposure and pre- and postnatal high-fat diet on systolic blood pressure (F = 22.686, df = 1, *p*<0.01) (Figure 1D).

### Body weight

Body weight of female offspring rats in LPS+High-fat group decreased significantly compared with Control group, LPS group and High-fat group (*p*<0.01) (Figure 1E); body weight in male offspring rats in both High-fat group and LPS+High-fat group decreased significantly compared with Control group and LPS group (*p*<0.01) (Figure 1F). There existed significant interaction between prenatal LPS exposure and pre- and postnatal high-fat diet on body weight of female offspring rats (F = 33.624, df = 1, *p*<0.01).

### Serum glucose and insulin concentration

**Figure 2:**
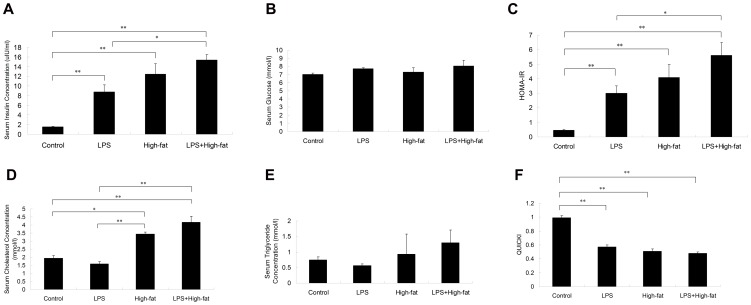
Effects of prenatal exposure to lipopolysaccharide combined with pre- and postnatal high-fat diet on serum insulin (A), glucose (B), HOMA-IR (C), serum cholesterol(D), serum triglyceride (E) and QUICKI (F) in three-month old offspring rats. Data are presented as the mean ± SEM (n = 8 in each group). ^**^ *P*<0.01, ^*^ *P*<0.05 (one-way ANOVA).

Serum insulin in LPS, High-fat and LPS+High-fat groups increased significantly compared with Control group (*p*<0.01). Compared with LPS group, serum insulin in the LPS+High-fat group also increased significantly (*p*<0.05) (Figure 2A). No significant difference was found among all groups in serum glucose (Figure 2B).

### Serum cholesterol and triglyceride concentration

Serum cholesterol concentration in High-fat group (*p*<0.05) and LPS+High-fat group (*p*<0.01) increased significantly compared with Control group and LPS group (*p*<0.01) (Figure 2C). There existed significant interaction between prenatal LPS exposure and pre- and postnatal high-fat diet on serum cholesterol concentration (F = 325.371, df = 1, *p*<0.01). There was no significant difference among all groups in serum triglyceride concentration (Figure 2D).

### HOMA-IR and QUICKI

Compared with Control group, HOMA-IR in LPS, High-fat and LPS+High-fat groups increased significantly (*p*<0.01) (Figure 2E). HOMA-IR in LPS+High-fat group also increased significantly compared with LPS group (*p*<0.05) (Figure 2E).

QUICKI in LPS group, High-fat group and LPS+High-fat group decreased significantly compared with Control group (*p*<0.01) (Figure 2F).

### Liver and renal function evaluation

Serum alanine aminotransferase concentration increased significantly in LPS+High-fat group compared with Control group (*p*<0.01). Serum aspartate aminotransferase concentration increased significantly in High-fat group (*p*<0.05) and LPS+High-fat group (*p*<0.01). No significant difference was found among all groups in serum total bilirubin, total protein and albumin concentration (Table 1).

**Table 1:** Effects of prenatal exposure to lipopolysaccharide combined with pre- and postnatal high-fat diet on liver and renal function in offspring rats.

	Control	LPS	High-fat	LPS+High-fat
Alanine aminotransferase (u/L)	29.88±3.74	29.75±2.89	39.00±6.00	74.75±22.32**
Aspartate aminotransferase (u/L)	87.00±7.93	94.75±6.35	211.50±32.50*#	228.75±49.56**##
Total bilirubin (umol/L)	0.55±0.11	0.34±0.11	0.35±0.25	0.13±0.06
Total protein (g/L)	75.38±3.42	69.25±0.79	71.00±1.00	73.00±1.63
Albumin (g/L)	41.13±1.30	39.50±0.60	42.00±1.00	36.75±1.38
Urea nitrogen (mmol/L)	5.85±0.48	5.93±0.49	8.65±1.15	4.30±0.45
Creatinine (mmol/L)	59.00±4.52	46.63±2.45	50.00±10.00	47.00±8.83
Uric acid (mmol/L)	34.25±5.77	44.63±5.14	96.50±6.50	53.50±13.38

* *p*<0.05 vs Control group; ^**^ *p*<0.01 vs Control group; ^#^ *p*<0.05 vs LPS group;

## *p*<0.01 vs LPS group.

No significant difference was found among all groups in serum urea nitrogen, creatinine and uric acid (Table 1).

### Morphological changes of thoracic aorta

**Figure 3:**
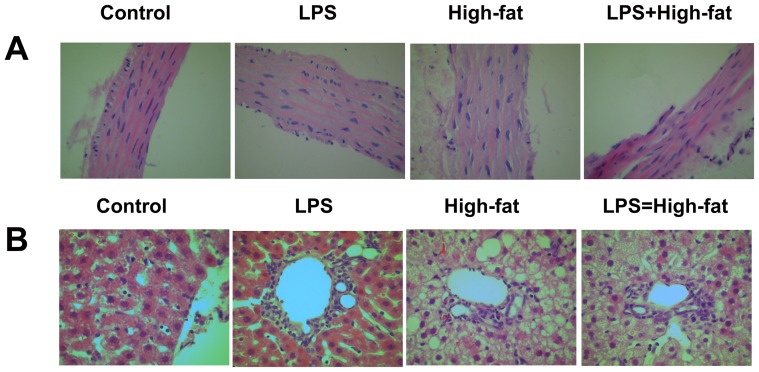
Prenatal exposure to lipopolysaccharide combined with pre- and postnatal high-fat diet result in morphological changes in thoracic aorta (A) and liver (B) tissue (H-E staining) in three-month old offspring rats.

Under light microscope, cells in thoracic aorta in Control group lined up in order without disruption, and the morphology structure of cells were normal, by contrast, the cells were disordered and loose, and with some deformed nucleus in LPS group and High-fat group (Figure 3A).

### Morphological changes of liver

Under light microscope, liver tissue in Control group was normal without inflammation and hepatocytes disruption, whereas in LPS group, macrophages and lymphocytes infiltration was found around central vein. Lipid accumulates in most of the hepatocytes as vacuoles with many nucleus disappeared in High-fat group and LPS+High fat group (Figure 3B).

## Discussion

The novel finding of this study was that prenatal exposure to LPS of 0.4 mg/kg resulted in hypertension and insulin resistance in offspring rats; prenatal exposure to LPS combined with pre- and postnatal high-fat diet resulted in lowered blood pressure, insulin resistance, lowered body weight and impaired liver function in offspring rats.

LPS acts as a non-specific immunostimulant to mimic the bacterial inflammatory response. It initiates a series of phosphorylation events by binding to Toll-like receptor 4 and promoting the translocation of nuclear transcription factor (NF)-κB into the nucleus, which promotes transcription of IL-6, IL-1β, and TNF-a, ultimately induces the inflammatory response [12]. Our previous study has showed that prenatal exposure to LPS (0.79 mg/kg) results in increased IL-6 and TNF-a concentration in amniotic fluid 12 h after intraperitoneal injection of LPS (mean value, LPS group vs control group, TNF-a concentration: 6.43 vs 3.18 fmol/ml; IL-6 concentration: 75 vs 50 pg/ml). We also found that prenatal LPS (0.79 mg/kg) exposure up-regulates IL-6 and TNF-a mRNA expression in fetus and causes hypertension in offspring rats [12], [13].

According to my previous studies, prenatal exposure to LPS of 0.79 mg/kg can cause abortion at a rate of 10% to 15%, therefore, 0.4 mg/kg would be safer. In the present study, we found that prenatal exposure to LPS of 0.4 mg/kg resulted in hypertension in three-month old offspring rats. Besides, Serum IL-6 and TNF-a increased significantly in High-fat group, which suggests an apparent inflammatory state created by high-fat diet. These findings can be supported by the previous studies [6], [12], [14].

In Kohmura's study, the pregnant mice were injected intravenously with 0.05 or 0.1 mg of LPS on day 14 to 16 of gestation. I-labeled LPS were injected into mice. Considerable amounts of the radioactivity were accumulated in the placenta and also in fetuses. This indicates that LPS can pass through the placenta and into fetuses. However, it is well-established that many of the biologic effects of LPS are mediated through the action of proinflammatory mediators released by host cells in response to LPS. These mediators including TNF-a, IL-1 and nitric oxide are mainly produced by macrophages [15]. In Ning's study, pregnant mice were injected intraperitoneally with a single dose of LPS (0.5 mg/kg) on gestational day 17. TNF-a obviously increased in maternal serum and amniotic fluid in response to LPS. When the pregnant mice were pretreated with a low-dose LPS (0.01 mg/kg, i.p.) at 4, 12, 24 or 48 h before LPS (0.5 mg/kg, i.p.), LPS-evoked TNF-a in maternal serum and amniotic fluid was significantly inhibited. Importantly, low-dose LPS pretreatment also greatly attenuated LPS-induced increases in TNF-a protein in fetal liver and fetal brain. Taken together, these results indicate that perinatal exposure to low-dose LPS induces a reduced sensitivity to subsequent LPS challenge [16]. Xu also found that, pretreatment with a low-dose LPS (0.01 mg/kg, i.p.) 24 h before high-dose LPS (0.12 mg/kg, i.p.) reduced sensitivity to subsequent high-dose LPS-induced intra-uterine fetal death, TNF-a production and oxidative stress in mice [17]. These can explain why prenatal exposure to LPS combined with high-fat diet result in normal level of serum TNF-a concentration in offspring rats.

Despite the hypertension or elevated blood pressure induced by prenatal exposure to LPS or high-fat diet, it was interesting to find that, a lowered blood pressure was found in LPS+High-fat group with lowered body weight both in male and female offspring rats. It seemed that prenatal LPS plus pre- and postnatal high-fat diet reversed hypertension caused by LPS. This phenomenon might due to the interaction between prenatal LPS treatment and high-fat diet. In the present study, the high-fat diet was given on the day when pregnancy was detected, that is, 8 days earlier than the intraperitoneal LPS injection during pregnancy. As has been demonstrated in Troseid's study that “An increase in plasma LPS occurs in healthy individuals after a high-fat meal” [4], therefore, we hypothesize that the reduced blood pressure in offspring rats of high-fat group might be the result of predictive adaptive response to inflammation caused by LPS and high-fat diet [6], [7]. What in accordance with the lowered blood pressure was the normal level of serum IL-6 and TNF-a in LPS+High-fat group, which further convinced the beneficial effect of prenatal LPS exposure combined with pre- and early postnatal high-fat diet on blood pressure in offspring rats.

It was unexpected to find that body weight of male offspring in High-fat group was lower compared with Control and LPS group. It might because that the male fetuses are more vulnerable to prenatal adverse environment [8]. This result can be supported by previous study by Makarova who found that, leptin injections to C57Bl mice on day 17 of pregnancy decreased body weight in both male and female offspring but inhibited the food intake and diet-induced obesity only in male offspring. The maternal effect was more pronounced in male offspring. Their result showed that hyperleptinemia during pregnancy has gender-specific long-term effects on energy balance regulation in progeny and does not predispose offspring to developing obesity [18]. In Sánchez's study, offspring of dams supplemented with olive oil, butter, or margarine during late pregnancy and lactation were fed with normal fat diet until 4-month-old, and then with high fat diet until 6-month-old. In this model, the offspring displayed a lower body weight in both genders and lower body fat only in males, and the mechanism is also related to leptin [19]. Leptin suppresses food intake and increases energy expenditure by enhancing thermogenesis and metabolic rate [14]. It is associated with body mass index and body fat in non-obese and obese subjects and in patients with T2DM [20]. In the present study, increased serum leptin level exhibited in high-fat group. Therefore, it is likely that, the reduction of body weight in male offspring in High-fat group might correlate with leptin level, while the reduction of body weight in LPS+High-fat group might result from the interaction between LPS and high-fat diet.

In addition, it was observed that a hyperinsulinemia, hypercholesterolemia, higher HOMA-IR and lower insulin sensitivity exhibited in offspring rats of LPS group, High-fat group and LPS+High-fat group, which suggest higher insulin resistance in these groups. Previous studies have demonstrated that maternal exposure to LPS of 0.79 mg/kg or high-fat diet results in insulin resistance in adult offspring rats [10], [11], [21]. If the lowered blood pressure in LPS+High-fat group is due to predictive adaptive response to prenatal inflammation, it seemed that it could not protect the offspring from insulin resistance. The possible mechanism need further study.

Serum levels of both alanine aminotransferase and aspartate aminotransferase evelated in LPS+High-fat group, and elevated aspartate aminotransferase was also found in High-fat group, which suggest impaired liver function [22]. The morphological changes in liver tissue also convinced this finding. In High-fat group and LPS+High-fat group, inflammation and serious liver steatosis was observed in the liver tissue. H-E staining of thoracic aorta also showed an impaired structure of thoracic aorta in LPS and High-fat group, but it seemed that it was less impaired in LPS+High-fat group. These morphological changes further convinced the findings in blood pressure and insulin resistance.

In conclusion, inflammation induced by prenatal exposure to LPS and pre- and postnatal high-fat diet might exert a predictive adaptive response which protects the offspring rats from hypertension; at the same time, the maternal metabolism might also be influenced, and in turn produced offspring with lowered body weight and insulin resistance. The further study will focus on whether prenatal exposure to LPS and High-fat diet cause change in maternal serum leptin level, and whether maternal exposure to LPS and high-fat diet only during pregnancy produce offspring without both hypertension and insulin resistance.
